# Type-2 immunity associated with type-1 related skin inflammatory diseases: friend or foe?

**DOI:** 10.3389/fimmu.2024.1405215

**Published:** 2024-05-29

**Authors:** Laure Migayron, Sylvie Bordes, Brigitte Closs, Julien Seneschal, Katia Boniface

**Affiliations:** ^1^ Univ. Bordeaux, CNRS, Immuno ConcEpT, UMR 5164, Bordeaux, France; ^2^ R&D Department, SILAB, Brive-la-Gaillarde, France; ^3^ CHU de Bordeaux, Dermatology and Pediatric Dermatology, National Reference Center for Rare Skin Disorders, Hôpital Saint-André, UMR 5164, Bordeaux, France

**Keywords:** type-1 immunity, type-2 immunity, atopic dermatitis, localized scleroderma, alopecia areata, vitiligo

## Abstract

Chronic inflammatory skin diseases are multifactorial diseases that combine genetic predisposition, environmental triggers, and metabolic disturbances associated with abnormal immune responses. From an immunological perspective, the better understanding of their physiopathology has demonstrated a large complex network of immune cell subsets and related cytokines that interact with both epidermal and dermal cells. For example, in type-1-associated diseases such as alopecia areata, vitiligo, and localized scleroderma, recent evidence suggests the presence of a type-2 inflammation that is well known in atopic dermatitis. Whether this type-2 immune response has a protective or detrimental impact on the development and chronicity of these diseases remains to be fully elucidated, highlighting the need to better understand its involvement for the management of patients. This mini-review explores recent insights regarding the potential role of type-2-related immunity in alopecia areata, vitiligo, and localized scleroderma.

## Introduction

The characterization of the diversity of immune cell subsets has extended our understanding of the complexity of the mechanisms driving the development and recurrence of chronic inflammatory disorders and hastened the subsequent use of targeted therapies. Three major types of innate and adaptive cell-mediated effector immunity have been identified (1, 2). While these immune responses are primarily involved in protection against pathogens, their aberrant activation can also be harmful and lead to the development of autoimmunity or to inflammatory or allergic diseases (1, 3).

Type-1 immunity mainly involves innate lymphoid type-1 cells (ILC1), natural killer (NK) cells, CD4 Th1 and cytotoxic CD8 Tc1 cells, mainly inducing interferon (IFN)-γ and tumor necrosis factor (TNF)-α (1). Besides its protective role against intracellular pathogens such as viruses, it is also implicated in inflammatory diseases such as alopecia areata (AA), localized scleroderma (LS), and vitiligo.

**Figure 1:**
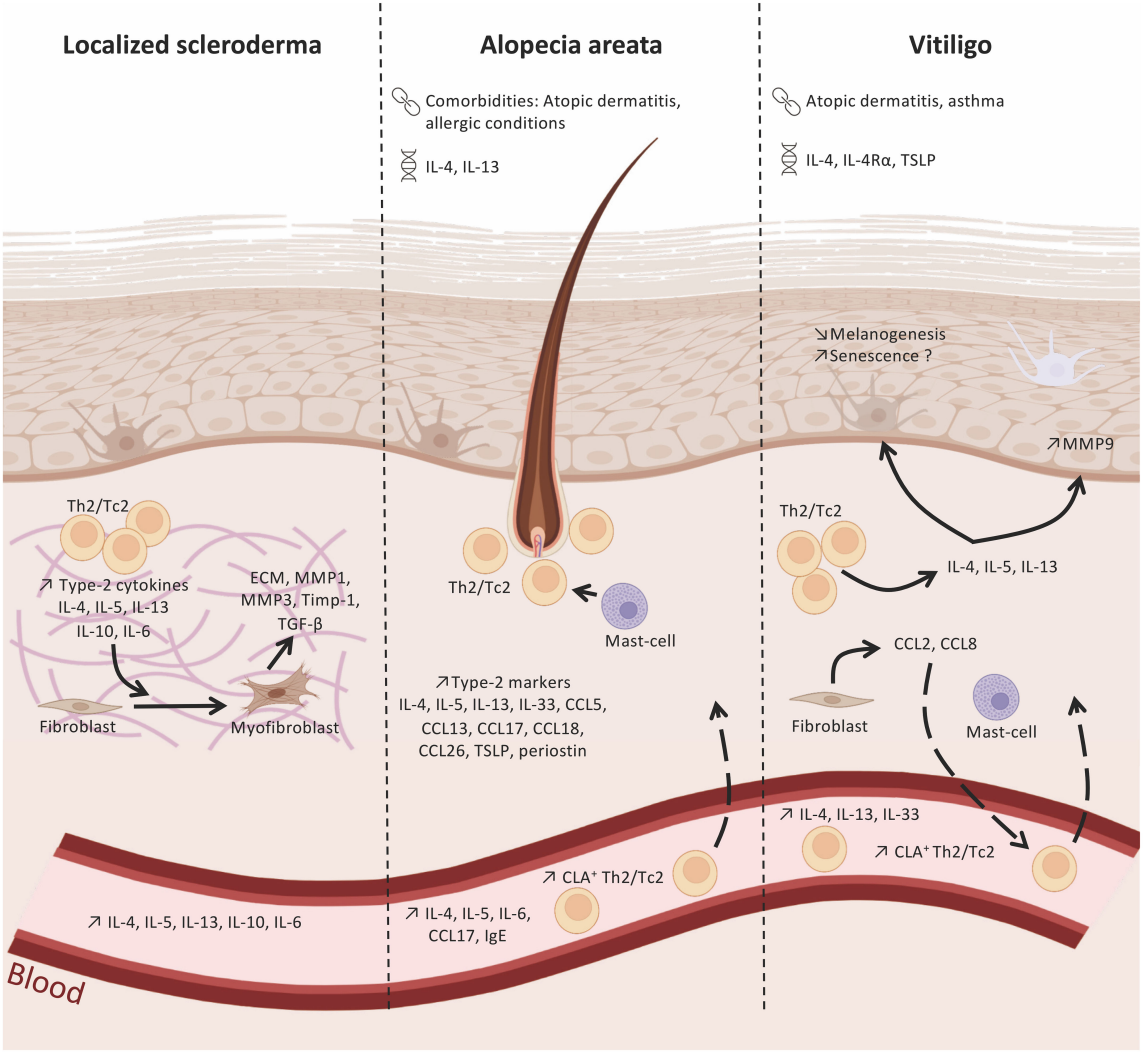
Type-2 immunity in localized scleroderma, alopecia areata and vitiligo. Polymorphims with IL-4/IL-13 genes have been identified in alopecia areata (AA) and vitiligo. In addition, these two diseases are associated with atopic dermatitis or allergic conditions. Type-2 immunity cells and markers found in the skin and blood of patients withlocalized scleroderma (LS), AA, and vitiligo, suggesting their role in the immune network of these pathologies. In LS, type-2 cytokines released by Th2 and Tc2 cell subsets (e.g. IL-4, IL-5, IL-13) infiltrating LS lesions promote the differentiation of fibroblasts into myofibroblasts and the production of pro-fibrotic factors, like TGF-β. AA skin lesions display elevated levels of type-2 cytokines and chemokines released by epidermal, dermal, and immune cells that will contribute to the recruitment of Th2/Tc2 and my influence hair loss. The type-2 environment in vitiligo skin may regulate melanogenesis and the loss melanocytes.
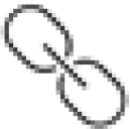
: comorbidities; 
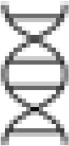
polymorphisms. Created with BioRender.com.

Type-2-associated immune cells include ILC2, Th2 cells, eosinophils, mast cells, basophils, and alternatively activated macrophages, which are known to release cytokines like interleukin (IL)-4, IL-5, IL-13, or IL-31 (3–6). These cells are critical for the defense of the organism against extracellular pathogens (i.e. helminth parasites) and for the maintenance of tissue homeostasis (tissue regeneration and wound repair) (7). However, besides its protective role, pathogenic activation of type-2 immune response contributes to the development of allergic and inflammatory diseases such as asthma, allergic rhinitis, and atopic dermatitis (AD) (5, 8, 9). AD is characterized by skin barrier dysfunction contributing to an aberrant sensitization to environmental allergens (10). In AD, type-2 cytokines like IL-4 and IL-13 are directly implicated in the impairment of epidermal barrier integrity observed in AD lesions by inhibiting the synthesis of key structural proteins such as filaggrin, loricrin, honerin, and involucrin (11–14). The role of type-2 inflammation in AD, and especially that of IL-4/IL-13, is exemplified by the efficacy of therapies targeting these cytokines, e.g. the anti-IL-4Rα antibody dupilumab and the anti-IL-13 antibodies tralokinumab and lebrikizumab for moderate to severe AD (15–30). However, the pathophysiology of AD is more complex with heterogenous phenotypes underlying different endotypes, with the involvement of type-1 (e.g. Th1 cells) and/or type-3 (Th17 and Th22 cells) immune cell subsets (31–35). Likewise, an increasing body of evidence has shown that type-2-associated immune response may also play a role in the development of type1 or type-3-related skin diseases, hence increasing the complexity of disease pathogenesis and patient stratification. This mini-review examines recent insights into the role of type-2 inflammation in type-1-associated skin inflammatory diseases with a focus on LS, AA, and vitiligo (**Figure 1**).

## Localized scleroderma

LS is a rare autoimmune skin disorder characterized by inflammation and fibrosis of the skin, with dense collagen deposition in the dermis and underlying connective tissues (36). Inflammatory patches and/or bands of thickened skin develop on the head and neck region, trunk and extremities. Morphea is the most frequent subtype of LS with onset between 40 and 50 years of age (37). LS is classified into five main types according to the extent and depth of fibrosis: limited, generalized, linear, deep and mixed (37, 38). Its pathogenesis is based on genetic predisposition combined with external triggers such as trauma, repeated friction, and surgery, that induce aberrant inflammatory and profibrotic responses, fibroblasts being a critical factor during the development of the disease (39–41). During the early inflammatory stage of LS, CD4^+^ T cells, macrophages, and eosinophils infiltrate the skin and adjacent blood vessels (36, 42, 43). Both Th1 and Th17 responses seem implicated in this primary stage, with an increased release of chemokine (C-X-C motif) ligand (CXCL)9/10, IFN-γ, TNF-α, IL-23, IL-17 and transforming growth factor (TGF)-β (36, 44). CXCL9 and CXCL10 serum levels correlate with the disease activity (45, 46). Interestingly, Werner et al. recently identified clusters of inflammatory fibroblasts prone to release CXCL12 or CXCL9/10 in LS lesions. The same study also demonstrated the crosstalk between fibroblasts and infiltrated immune cells (e.g. macrophages and T cells) to perpetuate inflammatory signals in lesions (47). Indeed, inflammatory fibrosis was shown to be dependent on CXCL9 and its receptor CXCR3 in a mouse model of skin fibrosis, thereby confirming the involvement of a type-1 immune response in the early phase of skin fibrosis (48). In addition, the increased expression of several adhesion molecules by endothelial cells, such as vascular cell adhesion molecule 1 (VCAM1), Intercellular Adhesion Molecule1 (ICAM1) and E-selectin, contributes to the recruitment of immune cells in the lesional areas (49).

Fibrosis is a key mechanism defining LS lesions and is characterized by excessive deposition of extracellular matrix (ECM) components such as collagen in the tissue. TGF-β is considered as a major profibrotic factor owing to its effects on fibroblast proliferation, differentiation, migration, and the production of extracellular cellular matrix components (50, 51). However, clinical trials blocking TGF-β produced conflicting results (52, 53).

It has been postulated that as the disease progresses, a shift occurs to a type-2 immune response that is associated with the development of skin fibrosis. Type-2-related cytokines (IL-4, IL-5, IL-6 and IL-13) are increased in the serum and skin of LS patients, and IL-13 serum levels correlate with the number of lesions in LS (54–56). Such type-2 immunity appears to be associated with the fibrotic/sclerotic stage of the disease (36). *In vitro* studies showed that IL-4 and IL-13 induce an excessive production of ECM components such as collagen, periostin, proteoglycan synthesis, and fibronectin by scleroderma and/or normal fibroblasts (57–61). These cytokines also stimulate the production of TGF-β and the synthesis of matrix metalloproteinase (MMP)1, MMP3 and TIMP-1 (a tissue inhibitor of MMP), as well as the proliferation of fibroblasts and their differentiation in myofibroblasts (62–64). Interestingly, the inhibition of type-2 signaling prevents the development of cutaneous fibrosis *in vivo* (65–67). A phase II clinical trial is ongoing to test the efficacy of dupilumab in localized scleroderma patients (NCT04200755).

## Alopecia areata

AA is a chronic non-scarring hair loss condition affecting 0.5–2% of the population and resulting from an autoimmune response targeting the hair follicle (68). AA is predominantly driven by a type-1 inflammatory response associated with the production of IFNγ by antigen-specific CD8^+^ NKG2D^+^ Tc1 and CD4^+^ Th1 cells in response to an environmental trigger, such as stress, viral infection, or trauma. This induces the collapse of the immune privilege of the hair follicle leading to its growth arrest (69). IFNγ also contributes to the increased inflammation through the induction of CXCL9/10 by the hair follicle epithelium, leading to the recruitment of CXCR3^+^ T cells to the bulb (70).

Recent data also suggest the contribution of the type-2 immune response in AA pathogenesis. From a clinical perspective, AA is associated with atopic dermatitis and allergic conditions, and an atopic background increases the risk of developing it (71–75). Loss-of-function mutations in the gene encoding filaggrin are associated with the severity of AA in patients with a history of AD, and genetic studies identified the association of AA with polymorphisms for the genes encoding IL-4 and IL-13 (76–78). An increase in mast cells with a pro-inflammatory phenotype in the perifollicular area of AA patients was reported. These mast cells display an increased degranulation activity and could interact with CD8^+^ T cells to provide co-stimulatory signals (via 4–1BBL, OX40L, ICAM1) and possibly to present neo-autoantigens (79). In addition, AA skin lesions display an increase in type-2-related cytokines and chemokines, including IL-4, IL-5, IL-13, IL-33, chemokine (C-C motif) ligand (CCL)-5, CCL13, CCL17, CCL18, CCL26, TSLP and periostin (80–83). Interestingly, after intralesional corticosteroid injection, a downregulation of CCL18 was associated with a clinical improvement (82). Levels of IL-4, IL-5, IL-6, IL-13, CCL13, CCL17, CCL22, CCL26, and IgE are also increased in AA patients’ sera (83–88). Czarnowicki et al. observed an increase in circulating skin-homing cutaneous lymphocyte-associated antigen (CLA)^+^ Th2 and CLA^+^ Tc2 cell subsets in AA patients compared to healthy controls that correlated with disease activity. In contrast, IFNγ was associated with the chronicity of the disease (89). All these data highlight the putative role of Th2 cells in disease pathogenesis.

However, the use of dupilumab in AA led to conflicting results, some investigations showing a significant improvement while others reporting exacerbation or new onset of the disease (90–100). Patients with an atopic background and high IgE levels exhibited a better response to dupilumab (101). Recent data suggest that non-atopic AA patients display an increase in circulating Tc1 cells while AA patients with concomitant AD show a skewed Th2 profile (102). In addition, the infiltration of CCR4^+^ Th2 cells around the hair bulb in skin lesions is more extensive in AA patients with AD (102). Altogether, these data suggest that as in AD, different clinical phenotypes and related endotypes likely define AA patients.

## Vitiligo

Vitiligo, the most common depigmenting skin disease, is defined by a type-1 skewed immune bias, with the involvement of IFNα, IFNγ, TNFα, CXCL9, CXCL10, and CXCL16 (103–105). Melanocyte loss results from the cytotoxic activity of CD8 T cells and detachment of melanocytes from the basal layer of the epidermis in response to the cytokine microenvironment (106). The perilesional skin of vitiligo patients is characterized by the infiltration of CXCR3^+^ NKG2D^+^ melanocyte-specific resident memory CD8 T cells and recirculating memory T cells producing IFNγ and TNFα (107–112). These type-1 cytokines impair the expression of genes involved in melanocyte adhesion, function, and melanogenesis (113, 114). IFNγ and TNFα also induce melanocyte detachment through the production of MMP9 by keratinocytes, which cleaves E-cadherin, a transmembrane glycoprotein important for melanocyte adhesion (115). In addition, these cytokines amplify the local inflammation through the release of CXCL9/10 by epidermal cells (105, 107). The type-1 inflammation is not restricted to the perilesional skin but concerns also the nonlesional skin of vitiligo patients (116).

Recent data suggest the involvement of a more complex cytokine network in disease pathogenesis with the involvement of type-2 cytokines. Epidemiological studies demonstrated the association of vitiligo with atopic diseases driven by a type-2 immune response, like AD or asthma (117–120). Genome wide association studies (GWAS) identified TSLP gene polymorphism in patients with vitiligo (121). Despite the absence of evidence from GWAS, smaller genetic studies identified polymorphisms of the gene coding for IL-4 as a risk factor for developing vitiligo (122, 123). These polymorphisms correlate with an increase in IL-4 and IgE levels in the serum of vitiligo patients. IL-4 receptor (IL-4R)-α and TSLP gene polymorphisms are associated with an increased susceptibility to vitiligo, reinforcing the putative role of type-2 cytokines in vitiligo (121, 124, 125). In addition, IL-4, IL-13, and IL-33 levels are increased in the serum of vitiligo patients (126–129). An increase in mast cells in vitiligo lesions was reported (130, 131). Czarnowocki et al. reported an increase in both circulating skin-homing CLA^+^ T cells producing IFNγ or IL-13 in patients. IL-13 levels decreased with vitiligo duration, suggesting its potential role in the early stages of the disease (132). We recently showed that vitiligo skin T cells produce both type-1 and type-2 cytokines, and in particular IL-13 (105). In addition, levels of chemokines that can be associated with a type-2 immune response, such as CCL5, CCL18, CXCL12, or CXCL16, are increased in vitiligo perilesional skin (104, 105, 133). A recent study in a mouse vitiligo model induced by the inoculation of melanoma cells, depletion of regulatory T cells, and excision of the tumor showed that IFNγ induces the secretion of CCL2 and CCL8 by dermal fibroblasts through JAK2/STAT1 signaling, resulting in type-2 cell attraction (134). Indeed, CCL2 is implicated in Th2 polarization and CCL8 in the recruitment of Th2 cells (135, 136). These data suggest the interconnected role of type-1 and type-2 immune responses in the inflammatory environment observed in vitiligo.

So far, the potential impact of type-2-related cytokines on melanocytes has received little attention. IL-4 and IL-13 were reported to inhibit melanogenesis (137, 138). Moreover, IL-13 induces the production of matrix metalloproteinase (MMP)-9 by keratinocytes (115, 139) and may therefore contribute to melanocyte loss in vitiligo together with IFNγ and TNFα. In addition, melanocytes and fibroblasts present a senescence pattern in vitiligo skin (140–142). IFNγ and TNF-α were shown to induce senescence in melanocytes (143, 144), and it would be interesting to evaluate the impact of type-2 cytokines in senescence in vitiligo given that IL-13 can promote senescence in submandibular glands (145). Nonetheless, type-2-related cytokines may also be protective in some subclinical subsets of vitiligo, since dupilumab induced or worsened vitiligo in AD patients (100, 146–148).

## Conclusion

Accumulating evidence is underlining the complexity of the cellular and cytokine network involved in the pathogenesis and flares of chronic autoimmune and inflammatory skin diseases. This diversity is likely linked to subclinical phenotypes and associated endotypes, as shown in AD. The role of the type-2 immune response is well characterized in atopic dermatitis and other type-2-related skin diseases. Recent data emphasize its role in other inflammatory skin disorders like vitiligo, AA and LS, which may explain the efficacy of small molecules like JAK inhibitors that target multiple cytokine pathways. In addition, the efficacy of emerging treatments targeting the type-2 response is being investigated, especially the IL-4/IL-13 axis in scleroderma and more recently in AA. The findings may be promising in a clinical subset of patients. Future studies will undoubtedly further decipher the role of the type-2 immune response in these diseases and provide insights into how they are involved in their pathogenesis and how to stratify patients. This may provide much needed guidance on choosing the most appropriate targeted therapy for patients.

## Author contributions

LM: Writing – original draft, Writing – review & editing. KB: Writing – original draft, Writing – review & editing. SB: Writing – review & editing. BC: Writing – review & editing. JS: Writing – original draft, Writing – review & editing.
